# Phenotypic Characterization of Intensive Care Patients With Infections: A Pilot Study of Host and Pathogen-Based Cluster Analysis

**DOI:** 10.7759/cureus.72255

**Published:** 2024-10-24

**Authors:** André Oliveira, Ana Rita Fernandes, Tânia F Mendes, João Gonçalves-Pereira

**Affiliations:** 1 Intensive Care Department, Hospital Vila Franca de XIra, Lisbon, PRT; 2 Critical Care Medicine, Universidade de Lisboa, Faculty of Medicine, Lisbon, PRT; 3 Internal Medicine Department, Hospital Vila Franca de Xira, Lisbon, PRT; 4 Intensive Care Department, Hospital Vila Franca de Xira, Lisbon, PRT

**Keywords:** cluster analysis, critical illness, infection focus, microorganism virulence, phenotypes, sepsis

## Abstract

Introduction: Sepsis is a prevalent, albeit complex, disorder among critically ill patients and a “one-size-fits-all” approach does not seem applicable. Host intrinsic characteristics and microorganisms’ particularities may influence response to therapy and outcomes. Attempting to group patients and microorganism characteristics may be an important step in developing and facilitating personalized infection treatment plans. This work intends to identify infected patients’ clusters using clinical data that includes infection determinants: the isolated pathogen and the site of infection.

Methods: In this retrospective analysis, we included patients with a microbiologically documented infection and non-infected controls. Patients admitted between January 2015 and December 2019 in the intensive care unit (ICU) were included (aged 17-95 years). Those with isolated microorganisms during their ICU stay were further analyzed using cluster analysis (hierarchical clustering and K-means; SPSS version 25.0). Four primary outcomes were addressed: ICU and hospital mortality rate and ICU and hospital length of stay (LOS).

Results: This study included 1,923 patients, of whom 721 (37.5%) had at least one microbiological isolate during their ICU stay. Patients with at least one isolate identification were older (mean age 67.7 years vs. 65 years; p < 0.001) and had a higher ICU and hospital mortality (20.3% vs. 24.3%, p = 0.041; 26.9% vs. 38.4%, p < 0.001), as well as a longer LOS (median hospital LOS 8 vs. 18 days; p < 0.001) than patients without microorganisms identified. Patients with at least one isolated microorganism were divided into five different clusters. Notable differences were found in their ICU and hospital trajectories between clusters.

Conclusion: The cluster analysis approach provided valuable insights into the complex interplay between bacterial virulence, infection site, and patient outcomes in critical care medicine. Patients infected with bacteraemia by Gram-positive bacteria (cluster 2) or Enterobacteriaceae (Cluster 5) and fungal isolation in respiratory samples (Cluster 3) should prompt more aggressive clinical interventions, as these patients are more prone to die in the hospital.

## Introduction

Sepsis and infection are common problems in the intensive care unit (ICU) setting [1]. Due to its complexity, the meaning of the word “sepsis” has been subject to frequent updates, adapting to our understanding of its pathophysiology [2-5].

Nowadays, and despite the evolution of modern therapeutic interventions, sepsis remains one of the main causes of morbidity and mortality in ICUs. An estimated 48.9 million incident cases of sepsis were recorded worldwide in 2017 and roughly 11 million sepsis-related deaths were reported, representing 19.7% of all global deaths [3]. In a recently published Portuguese study, sepsis was diagnosed in 30.8% of patients admitted to an ICU. Of these, 24.1% died during the first month after ICU admission, reaching 41.8% after one year of follow-up [6]. Thus, in 2017, the World Health Organization recognized sepsis as a global health priority [7].

Moreover, infection and sepsis are largely heterogeneous in critically ill patients, due to many different variables, both intrinsic and extrinsic [8]. Specific host characteristics such as age, sex, medication, comorbidities, and diseases (especially those that interfere with innate and adaptive immunity), as well as genetic predisposition, play a fundamental role [9]. The pathogenic microorganisms, alongside the source and site of the infection, the inoculum, and the susceptibility to antibiotics also play a role [1]. Different microorganisms are equipped with distinct mechanisms (virulence factors) that allow evasion from the host’s innate immune system and cause different patterns of disease [10]. Recognizing and addressing this heterogeneity is paramount to the development of individualized optimal treatment strategies and improving patient outcomes [8].

Attempting to group patients and microorganism characteristics may be an important step in developing and facilitating personalized infection treatment plans. The use of machine learning phenotyping, to identify closed linked sub-groups of patients that may benefit from specific therapeutic approaches [11], may provide valuable insights into the complex interplay between bacterial virulence, infection site, and patient outcomes in critical care medicine.

Cluster analysis is one of the types of unsupervised machine learning tools that have been recruited to unravel patient heterogeneity [9]. Critically ill patients constitute one of the most heterogeneous populations in the hospital; therefore, identifying subgroups of intensive care unit patients with similar clinical needs may provide a framework for more efficient ICU care and target therapies, particularly in infection and sepsis [12].

## Materials and methods

In this retrospective analysis, we evaluated all patients with a microbiologically documented infection. A group of non-infected patients admitted during the same timeframe, serve as controls. In this pilot study, we address the feasibility of evaluating the interplay of the isolated microorganism, the site of infection, and the patient’s age. Patient data and bacterial isolates were used to perform cluster analysis, providing information about groups and pattern recognition of the different infections and their prognosis. According to our sample size, we select only those variables directly associated with infection, including the microorganism and the host.

Data from patients admitted between January 2015 and December 2019 in the Intensive Care Department of Hospital Vila Franca de Xira in Portugal were analyzed. All adult patients (>18 years) consecutively admitted were included in the study. Patients with microbiological isolates during their ICU stay were evaluated by cluster analysis and were considered “infected." We excluded subjects younger than 18 years or with an ICU length of stay (LOS) of less than one day, those with significantly incomplete data, or with samples considered contaminations, and patients participating in other studies. Each patient could only be included once. The study protocol was approved by the Hospital de Vila Franca de Xira Ethical Board (approval number 15/2024). Informed consent was waived due to the retrospective nature of the study and the fact that only aggregated data was evaluated, with no possible individual patient identification.

Demographic (age, gender), severity (Simplified Acute Physiology Score (SAPS) II), microbiological identification, and therapeutic (invasive ventilation, renal replacement therapy and vasopressor) data were collected. The type of admission to the ICU (either medical or surgical, scheduled, or unscheduled) was also registered. Clinical severity was assessed through the SAPS II. The need for invasive organ support therapy (invasive mechanical ventilation, renal support therapy, vasopressors) was evaluated. All microbiological isolates were evaluated and categorized according to their prevalence, and characteristics using VITEK (Biomerieux) and MALDI-TOF. As previously published [1], microorganisms were classified into fungi and bacteria; the bacterial isolates were divided into groups according to bacterial species and Gram test in 15 groups.

Statistical analysis

Descriptive statistics were calculated. Data were summarized as mean ± standard deviation or median (percentiles 25 and 75), according to data distribution. Normal distribution was evaluated by graphical analysis. Categorical variables were described as absolute numbers (%). The chi-square test was used to compare categorical variables, while continuous variables were evaluated with the Student T test or the Mann-Whitney U test, according to data distribution.

Clusters were calculated for patients with isolated microorganisms. For the generation of clusters, we used three different variables: patients’ age, subdivided into four categories (<50, 50-65, 66-80, >80 years); infection focus, according to the product of the isolated microorganism (abdominal products, respiratory samples, blood samples, cerebrospinal fluid, skin, and urinary samples); and isolated microorganism, categorized into 15 different sub-groups.

Cluster hierarchy was used to determine the number of clusters, according to Ward’s method. Ward’s method was chosen to be the one with more power to discriminate the subgroups after defining the distance interval through squared Euclidian for every case. After establishing the number of clusters, patients were classified into one cluster, through K-means clustering, an unsupervised learning algorithm. Variables were described for each cluster, using median and (percentiles 25 and 75) for quantitative variables and frequency and percentage for qualitative variables.

Outcomes were calculated for each cluster and the remaining population (without isolated microorganisms). Four primary outcomes were assessed: ICU and hospital mortality and ICU and hospital LOS. The need for invasive organ support (invasive ventilation, renal replacement therapy, and vasopressor) was measured for each cluster and the non-infected group. Statistical analysis was performed using IBM SPSS Statistics, version 29.0 (IBM Corp., Armonk, NY). All statistics were two-tailed, and the significance level was p < 0.05.

## Results

Demographic analysis and population characterization

We evaluated 1,963 patients admitted to the Hospital Vila Franca de Xira ICU Department, of whom 721 (37.5%) had at least one microbiological isolate during the ICU stay. The clinical and demographic data of the included patients are presented in Table 1, according to the presence of infection. Of note, infected patients were older and had a higher SAPS II score. Both the ICU and hospital mortality and LOS were significantly higher in infected patients. Moreover, invasive organ support therapy was more often required by infected patients (Table 1).

**Table 1:** Demographic and clinical characteristics SAPS II: Simplified Acute Physiology Score II; CRF: chronic renal failure; COPD: chronic obstructive pulmonary disease; ICU: intensive care unit; RRT: renal replacement therapy; * chi-square test; ** Students’ T test; $: Mann-Whitney U test

Sample characteristics	All patients (N = 1,963)	Non-infected (N = 1,242)	Infected (N = 721)	p-value^*^
Age (years)	66.0 ± 15.4	65.0 ± 16.2	67.7 ± 13.8	<0.001^**^
Male (N (%))	1143 (58.2)	710 (57.2)	433 (60.1)	0.217
SAPS score II	45.5 ± 19.6	42.7 ± 19.6	50.1 ± 18.7	<0.001^**^
Type of admission (N (%))				
Medical	1455 (74.3)	952 (77)	503 (69.8)	<0.001
Unscheduled surgery	308 (15.7)	142 (11.5)	166 (23)	
Scheduled surgery	194 (9.9)	142 (11.5)	52 (7.2)	
Comorbidities (N (%))				
Diabetes mellitus	446 (22.7)	286 (23)	160 (22.2)	0.696
Hypertension	609 (31)	376 (30.3)	233 (32.3)	0.362
Heart failure	233 (11.9)	150 (12.1)	83 (11.5)	0.772
CRF	221 (11.3)	136 (11)	85 (11.8)	0.571
COPD	82 (4.2)	55 (4.4)	27 (3.7)	0.486
Organ support and outcome				
Invasive ventilation (N (%))	962 (49)	525 (42.3)	437 (60.6)	0.036
Invasive ventilation median days (percentiles 25 and 75)	3 (2-5)	2 [2-4]	4 (2-7)	<0.001^$^
Vasopressor (N (%))	867 (44.2)	437 (35.2)	430 (59.6)	<0.001
RRT (N (%))	494 (25.2)	260 (20.9)	234 (32.5)	<0.001
ICU mortality (N (%))	427 (21.8)	252 (20.3)	175 (24.3)	0.041
Hospital mortality (N (%))	609 (31.1)	333 (26.9)	276 (38.4)	<0.001
ICU length of stay median days (percentiles 25 and 75)	2.7 (1.3-4.6)	2.1 (1.1-3.7)	3.9 (1.8-7.1)	<0.001^$^
Hospital length of stay median days (percentiles 25 and 75)	16.7 (5-20)	8 (4-14)	18 (8-34)	<0.001^$^

In Table 2, we present A) the focus of infection and B) all the isolated microorganisms. Urinary and blood samples were the most prevalent, followed by respiratory tract samples. Gram-positive were identified in 25.1% of samples. The most common isolated microorganisms were Gram-negative, especially *Escherichia coli* (23.4%) and *Klebsiella pneumoniae* (13.6%), while *Staphylococcus aureus* (9.6%) was the most prevalent Gram-positive bacteria. Fungal were isolated in 5.4% of patients.

**Table 2:** Global distribution of isolated microorganisms and positive microbiological samples Data presented as absolute number (%).

Microbiological samples	N (%)
Site of infection	
Respiratory	108 (15.0)
Abdominal	34 (4.7)
Blood	178 (24.7)
Urinary	203 (28.2)
Skin discharge	141 (19.6)
Intravenous central line	29 (4.0)
Others	28 (3.9)
Microorganism	
Gram-positive	
*Staphylococcus aureus*	69 (9.6)
*Staphylococcus epidermidis*	47 (6.5)
*Streptococcus pneumoniae*	24 (3.3)
*Enterococcus faecium*	10 (1.4)
*Enterococcus spp*.	59 (8.2)
Other gram-positive	31 (4.3)
Gram-negative	
*Acinetobacter baumannii *complex	2 (0.4)
*Enterobacter spp*.	27 (3.7)
*Escherichia coli*	169 (23.4)
*Klebsiella pneumoniae*	98 (13.6)
*Klebsiella spp.*	7 (1.0)
*Proteus spp.*	39 (5.4)
*Pseudomonas aeruginosa*	60 (8.3)
Other gram-negative	40 (5.5)
Fungal	
*Candida *spp.	39 (5.4)
Total	721

Cluster analysis and cluster description

**Figure 1:**
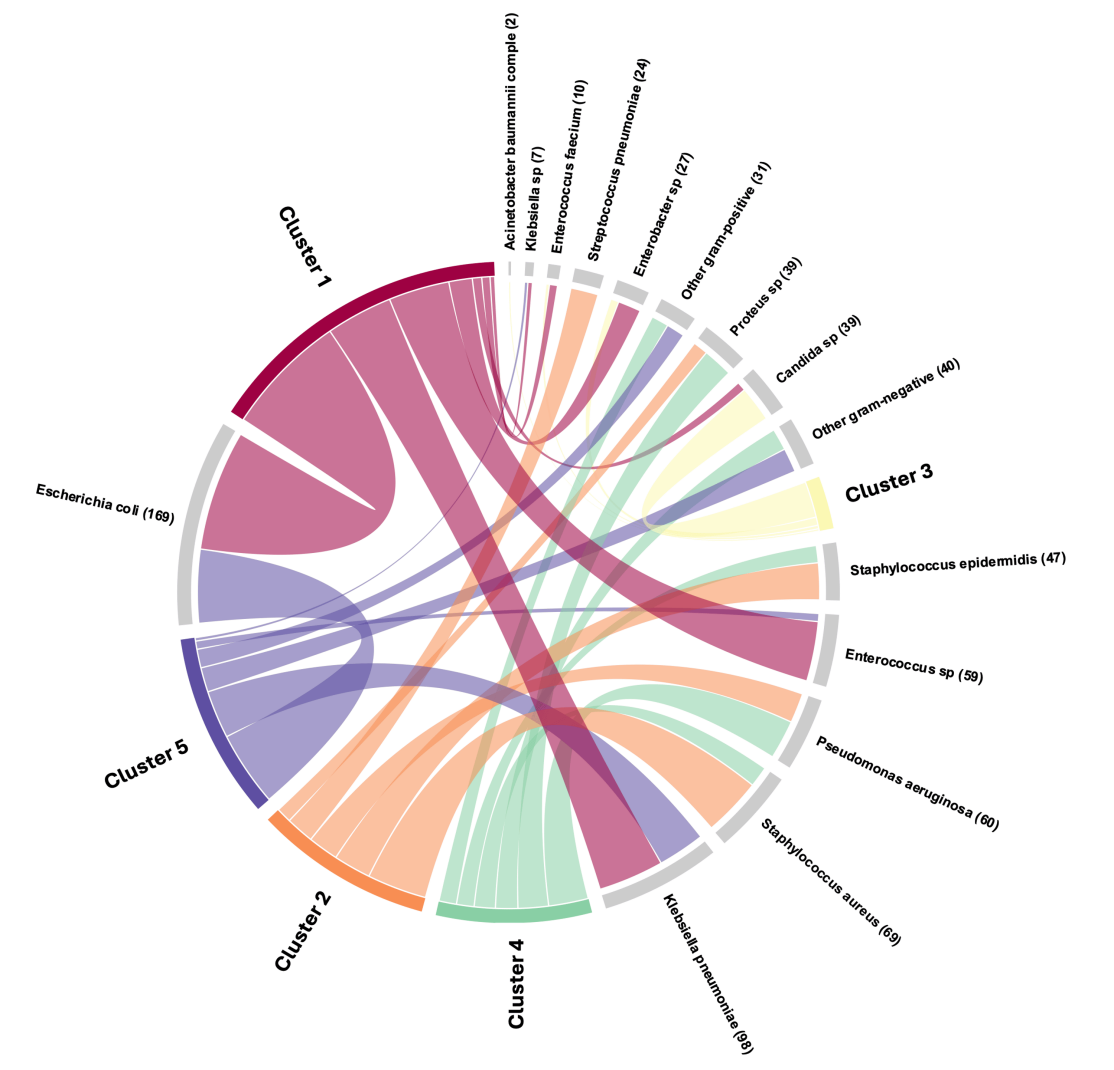
Pathogen by cluster Chord diagram, representing the cluster distribution per pathogen. Graphic made with RAWGraphs [13].

Five clusters were identified. In Table 3 and Figure 1, their main demographic characteristics, comorbidities, isolated microorganisms, and the respective sample, along with clinical and outcome data, are presented.

**Table 3:** Clusters characteristics SAPS II: Simplified Acute Physiology Score II; CRF: chronic renal failure; COPD: chronic obstructive pulmonary disease; ICU: intensive care unit; H: hospital; RRT: renal replacement therapy.; * chi-square test; ** Students’ T test; $ Mann-Whitney U test

Characteristics	Cluster 1 (N = 253)	Cluster 2 (N = 146)	Cluster 3 (N = 43)	Cluster 4 (N = 127)	Cluster 5 (N = 152)	p^*^
Host characteristics						
Age (years)	68.0±13.7	65.7±14.1	69.3±13.5	68.1±13.6	68.2±13.6	0.373^**^
Male (%)	136 (53.8)	91 (62.3)	28 (65.1)	84 (66.1)	94 (61.8)	0.129
SAPS score II	48.5±18	48.6±17.2	56.3±19.7	50±19.6	52.4±19.7	0.055^**^
Type of admission (%)						
Medical	157 (62.1)	134 (91.8)	24 (55.8)	75 (59.1)	113 (74.3)	<0.001
Unscheduled surgery	69 (27.3)	11 (7.5)	17 (39.5)	34 (26.8)	35 (23)	
Scheduled surgery	27 (10.7)	1 (0.7)	2 (4.7)	18 (14.2)	4 (2.6)	
Comorbidities (%)						
Diabetes mellitus	64 (25.3)	29 (19.9)	7 (16.3)	28 (22)	32 (21.1)	0.582
Hypertension	91 (36)	44 (30.1)	11 (25.6)	45 (35.4)	42 (27.6)	0.304
Heart failure	29 (11.5)	22 (15.1)	2 (4.7)	16 (12.6)	14 (9.2)	0.315
CRF	36 (14.2)	20 (13.7)	3 (7.0)	12 (9.4)	14 (9.2)	0.336
COPD	7 (2.8)	7 (4.8)	5 (11.6)	6 (4.7)	2 (1.3)	0.023
Organ support and outcome						
Ventilation N (%)	139 (54.9%)	90 (61.6%)	34 (79.1%)	76 (59.8%)	98 (64.5%)	0.032
Invasive ventilation median days (percentiles 25 and 75)	3 (2-6)	4 (2-8)	6 (3-9)	3 (2-7)	3 (2-7)	0.047^$^
RRT (%)	76 (30)	44 (30.1)	18 (41.9)	40 (31.5)	56 (36.8)	0.386
Vasopressor (%)	124 (49)	83 (56.8)	37 (86)	73 (57.5)	113 (74.3)	<0.001
ICU mortality (%)	37 (14.6)	45 (30.8)	16 (37.2)	18 (14.2)	59 (38.8)	<0.001
H mortality (%)	77 (30.6)	63 (43.2)	23 (53.5)	36 (28.3)	77 (51)	<0.001
ICU length of stay median days (percentiles 25 and 75)	3.9 (2.0-6.6)	3.9 (1.8-7.9)	4.7 (2.8-8.7)	3.9 (1.8-7.7)	3.7 (1.6-6.5)	0.242^$^
H length of stay median days (percentiles 25 and 75)	19 (10-37)	13.5 (6-28)	16 (8-28)	26 (15-48)	11 (6-25)	<0.001^$^
Microorganism N (%)						
*Acinetobacter baumannii *complex	0	0	2 (4.7)	0	0	<0.001
*Candida* spp.	8 (3.2)	0	31 (72.1)	0	0	<0.001
*Enterobacter* spp.	20 (7.9)	0	7 (16.3)	0	0	<0.001
Enterococcus faecium	7(2.8)	0	3 (7.0)	0	0	<0.001
*Enterococcus* spp.	52 (20.6)	0	0	0	7 (4.6)	<0.001
Escherichia coli	105 (41.5)	0	0	0	64 (42.1)	<0.001
Klebsiella pneumoniae	57 (22.5)	0	0	0	41 (27.0)	<0.001
*Klebsiella* spp.	4 (1.6)	0	0	0	3 (2.0)	<0.001
Other gram-negative	0	0	0	19 (15.0)	21 (13.8)	<0.001
Other gram-positive	0	0	0	15 (11.8)	16 (10.5)	<0.001
*Proteus* spp.	0	13 (8.9)	0	26 (20.5)	0	<0.001
Pseudomonas aeruginosa	0	26 (17.8)	0	34 (26.8)	0	<0.001
Staphylococcus aureus	0	51 (34.9)	0	18 (14.2)	0	<0.001
Staphylococcus epidermidis	0	32 (21.9)	0	15 (11.8)	0	<0.001
Streptococcus pneumoniae	0	24 (16.4)	0	0	0	<0.001
Sample type N (%)						
Respiratory	0	52 (35.6)	21 (48.8)	0	35 (23.0)	<0.001
Abdominal	0	4 (2.7)	6 (14.0)	0	24 (15.8)	<0.001
Blood	0	82 (56.2)	8 (18.6)	0	88 (57.9)	<0.001
Skin discharge	78 (30.8)	1 (0.7)	7 (16.3)	55 (43.3)	0	<0.001
Urine	158 (62.5)	0	0	45 (35.4)	0	<0.001
Catheter-related	11 (4.3)	0	0	18 (14.2)	0	<0.001
Others	6 (2.4)	7 (4.8)	1 (2.3)	9 (7.1)	5 (3.3)	<0.001

Cluster 1 (N = 253, 35.1%)

Most patients were included in this cluster. A higher prevalence of comorbidities, especially arterial hypertension (36%) and diabetes mellitus (25.3%) were noted. Enterobacterales were the most common isolated pathogen (65.6%), recovered mostly from urinary samples (62.5%). This cluster had the lowest SAPS II score (48.5±18.0) and relatively low mortality (ICU 14.6%; in-hospital 30.6%).

Cluster 2 (N = 146, 20%)

This cluster comprised the youngest patients (median age 65.7 ±14.0), who were admitted mostly for medical reasons (91.8%). The majority of the isolated pathogens were recovered from blood samples (56.2%). Gram-positive bacteria largely predominated (73.3%), particularly *Staphylococcus aureus* (34.9%).

Cluster 3 (N = 43, 6.0%)

This was the smallest cluster. It gathered the oldest patients (69.3 ± 13.5), of which 11.6% had chronic obstructive pulmonary disease. Samples came mostly from the respiratory tract (48.8%) and included 79.5% of all isolated *Candida *spp. These patients more often received invasive mechanical ventilation and were the group with the longest duration of vasopressor therapy. Not surprisingly, this cluster also had the highest SAPS II score (56.3 ± 19.7) and the highest in-hospital mortality (53.5%).

Cluster 4 (N = 127, 17.6%)

Patients in this cluster had the lowest in-hospital mortality (28.3%) despite having a longer median of hospital LOS (26 days). Most microorganisms were isolated from purulent skin discharge (43.3%) or intravenous central line (14.2%). The most common isolated pathogen was *Pseudomonas aeruginosa* (26.8%), followed by *Proteus* spp. (20.5%).

Cluster 5 (N = 152, 21.1%)

Alongside cluster 2, most microorganisms in this cluster were isolated from blood cultures, although mostly were Gram-negative (62.3%). The use of vasopressors was very common (74.3%). This was the cluster with the highest ICU mortality rate (38.8%), and more than half of these patients did not survive to hospital discharge.

**Figure 2:**
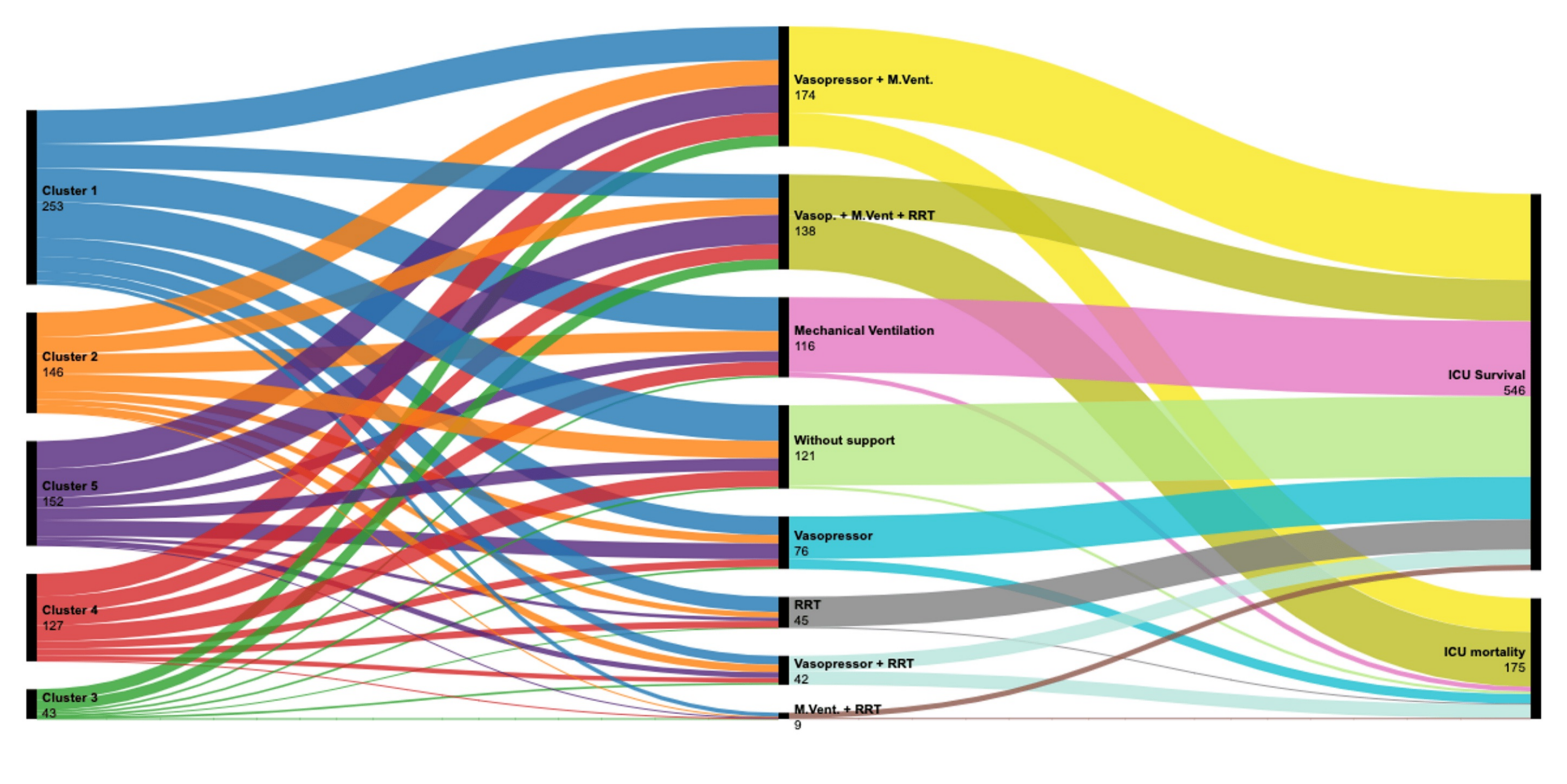
Sankey diagram Sankey diagram, presenting each cluster trajectory. RRT: renal replacement therapy; ICU: intensive care unit; M. Vent.: mechanical ventilation. Graphic made with RAWGraphs [13].

The different clusters' ICU trajectories can be better exposed through a Sankey diagram, showing various levels of organ support combination and distinctive outcomes (Figure 2).

## Discussion

In this study, we evaluated 1,923 patients and identified 721 (37.5%) with at least one microbiological isolate. These patients were significantly older and had more severe disease than the non-infected population. Their mortality was higher, both in the ICU (24.3 vs. 20.3%, p = 0.041) and, particularly, in the hospital (38.4 vs. 26.9%, odds ratio 1.7, 95% CI 1.4-2.07, p < 0.001). Both ICU and hospital LOS were also longer in infected patients.

It was possible to include the pathogen and the sample from where it was isolated, alongside the patient’s age, in a machine-learning model to identify different clusters, with different intra-hospital trajectories.

It is important to note that our definition of infection requires a microbiological isolate. It is well-known that several patients with community-acquired sepsis do not have microbiological isolates, especially with community-acquired pneumonia [14] and, consequently, a lower prevalence of infection was to be expected. Accordingly, we also note a higher rate of urinary isolates, while in most international epidemiological studies [1,15], respiratory infections are largely predominant, followed by bloodstream and abdominal infections.

Nevertheless, in all these studies, infection was always associated with higher mortality, and the foci of infection should play a role [1,15,16]. Sepsis, defined as a dysregulated and mal-adaptative host response to an infection remains one of the leading causes of mortality, especially in the ICU. However, due to its complex pathophysiology and great heterogeneity both in terms of clinical expression, and patient response to therapeutic interventions, sepsis continues to be incompletely understood. This heterogeneity is a barrier to effective therapeutic approaches. Clinical trials often address heterogeneous populations. However, the absence of benefit of a particular intervention to the general population may not exclude a clinical benefit from a (albeit small) group of patients. Identification of clinical phenotypes, using machine learning, may be a way to overcome this predicament.

In our study, we were able to divide the infected population into five different clusters. The clusters were largely independent, with different patient trajectories and hospital mortality rates. Unveiling differences in severity and prognosis may help to allocate resources better, namely, vigilant monitoring, and aggressive therapeutic interventions (Table 3, Figure 2).

The isolation of *Candida* in the respiratory samples in Cluster 3 may be related to the host immunosenescence and not to the infection itself [17]. Consequently, this could be a marker of frailty or poor physiological reserve. This could lead to poor outcomes independently of the infection itself. This highlights the importance of addressing these issues, the bug and the host, instead of analyzing only one specific aspect.

Targeting specific groups of patients to special therapeutic interventions has already been done. In the Prowess trial, the clinical benefits of *Drotecogin alfa* were considered more straightforward in the more severe groups (with an APACHE II score over 24) and only approved for this group [18]. Nevertheless, a second trial failed to show this benefit [19]. More recently, several COVID therapies were segregated to patients according to respiratory disease and the need for oxygen supplementation [20,21].

Nevertheless, this kind of patient selection is largely based on one single characteristic that may not have the same impact on different patients. Machine learning can analyze multiple patient characteristics and their interaction in a much shorter time frame, allowing a true personalization of therapy [22]. The promise of precision medicine is a greater understanding of individual sepsis clinical expression, and heterogeneous treatment response patterns will lead to "personalized" medicine and improved outcomes.

Consequently, defining clusters may allow the study and identification of patients who might benefit from controversial interventions (and those who might not). For instance, the use of one or two antibiotics; the early initiation of renal replacement therapy; the use of corticosteroids; the type of vasopressors combination; and the use of deeper sedation during invasive mechanical ventilation.

One of the greatest limitations of this study and our approach is the time needed to have a positive culture. Nevertheless, several microbiological techniques are now available, which should facilitate early recognition of the microorganism [23]. We recognize that the inclusion of newly found microorganisms (like viruses) may considerably change our results. Furthermore, we cannot exclude that some patients colonized (not infected) were misclassified. Moreover, we acknowledge that our cohort was small for this type of analysis and, as a single-center study, external validation is needed. Future studies need to be conducted at international consortiums so that a larger population of infected patients can be characterized.

Moreover, some patients might have been colonized and not infected, and infected patients with negative cultures were excluded. Finally, due to its retrospective nature, this study may suffer from biased missingness and imprecision in measurements related to clinical rather than research measurements. Due to the design of the study, we did not gather information for out-of-hospital mortality, which may contribute to a better understanding of prognosis.

This study pretends to be a “proof-of-concept” analysis. We believe that identifying the relationship between the bug and the host (both age and infection focus) will lead to better therapeutical decisions and resource allocation. Our ability to identify clusters with different trajectories seems to unveil that using microbiological and host information, personalized therapeutic interventions may be possible.

## Conclusions

Patients with sepsis and microbiologically documented infections can be divided into sub-groups (clusters), according to the identified microorganism and patients’ characteristics.

In the present study, five distinct clusters of critically ill septic patients were identified, with distinct clinical trajectories. For instance, patients infected with bacteremia by Gram-positive bacteria (Cluster 2) or Enterobacteriaceae (Cluster 5) and fungal isolation in respiratory samples (Cluster 3) should prompt more aggressive clinical interventions, as these patients are more prone to die in the hospital.

This provides valuable insights into the complex interplay between age, bacterial virulence, source of infection, and patient outcomes in critical care medicine. Understanding infection and sepsis using all these variables together may lead to a more complete understanding than using these factors independently. This approach may be used in future studies to support tailored research and clinical therapies for septic patients.
